# Epothilones Suppress Neointimal Thickening in the Rat Carotid Balloon-Injury Model by Inducing Vascular Smooth Muscle Cell Apoptosis through p53-Dependent Signaling Pathway

**DOI:** 10.1371/journal.pone.0155859

**Published:** 2016-05-24

**Authors:** Dong Ju Son, Jae Chul Jung, Jin Tae Hong

**Affiliations:** 1 College of Pharmacy and Medical Research Center, Chungbuk National University, Cheongju, Chungbuk, Korea; 2 Institute of Life Science Research, Rexgene Biotech Co., Cheongju, Chungbuk, Korea; University of Sassari, ITALY

## Abstract

Microtubule stabilizing agents (MTSA) are known to inhibit vascular smooth muscle cell (VSMC) proliferation and migration, and effectively reduce neointimal hyperplasia and restenosis. Epothilones (EPOs), non-taxane MTSA, have been found to be effective in the inhibition of VSMC proliferation and neointimal formation by cell cycle arrest. However, effect of EPOs on apoptosis in hyper-proliferated VSMCs as a possible way to reduce neointimal formation and its action mechanism related to VSMC viability has not been suited yet. Thus, the purposes of the present study was to investigate whether EPOs are able to inhibit neointimal formation by inducing apoptosis within the region of neointimal hyperplasia in balloon-injured rat carotid artery, as well as underlying action mechanism. Treatment of EPO-B and EPO-D significantly induced apoptotic cell death and mitotic catastrophe in hyper-proliferated VSMCs, resulting in cell growth inhibition. Further, EPOs significantly suppressed VSMC proliferation and induced apoptosis by activation of p53-dependent apoptotic signaling pathway, Bax/cytochrome c/caspase-3. We further demonstrated that the local treatment of carotid arteries with EPOs potently inhibited neointimal lesion formation by induction of apoptosis in rat carotid injury model. Our findings demonstrate a potent anti-neointimal hyperplasia property of EPOs by inducing p53-depedent apoptosis in hyper-proliferated VSMCs.

## Introduction

Percutaneous-transluminal-coronary-angioplasty (PTCA) with stent placement is the standard strategy to treat coronary artery disease but, neointimal hyperplasia with resultant restenosis following interventional procedure remains the major limitation in the clinical treatment [1–4]. Therefore, neointimal hyperplasia is a key mechanism that decreases late PTCA patency. Although neointimal hyperplasia is a complex process and that precise molecular mechanisms remain unclear, many studies have documented that proliferation and migration of the vascular smooth muscle cell (VSMC) plays a key role in the process of restenosis following intervention [5–8]. It is also well documented that apoptosis of VSMCs is another important regulator to the neointima formation [9, 10]. Abnormal tissue growth depends on the delicate balance between cell proliferation and apoptosis. It has been demonstrated that increasing VSMCs apoptosis could decrease neointimal hyperplasia [11, 12]. Further, prior efforts to reduce the extent of restenosis have focused on means of reducing the proliferation and migration of VSMCs or of increasing their apoptosis [13]. Therefore, much attention has been devoted to develop ways that regulates VSMC function and survival to prevent neointima formation.

In order to minimize restenosis rate by inhibiting VSMC proliferation and migration, advanced therapeutic approaches, including the use of drug-eluting stents (DES) and drug-coated balloons (DCB), have been evolving rapidly and show the potential efficacy in clinical settings [4, 14]. In the nineties, Paclitaxel (PTX), a microtubule stabilizing agent (MTSA) of the taxane family, was found to inhibits VSMC proliferation and migration and effectively reduces neointimal hyperplasia and restenosis [15, 16]. Further, PTX has been used as the primary drug for DEB and DCB because of its rapid uptake and prolonged retention until the present day [17, 18]. Epothilones (EPOs) are a novel class of non-taxane MTSAs, originally identified as metabolites of myxobacterium *Sorangium cellulosum*. The action mechanism of EPOs is similar to taxanes, but with more potent antiproliferative and anticancer activities [19–22]. In addition, it is also well known that EPOs induce apoptotic cell death in multiple cancer cells [23–25]. Previously, EPOs have been discovered that inhibit neointimal formation after balloon-injury in rat carotid artery by inhibition of VSMC proliferation through cell cycle arrest [26, 27]. However, pro-apoptotic effect of EPOs in hyper-proliferated VSMCs as a possible way to reduce restenosis rate after angioplasty intervention and its action mechanism related to VSMC viability has not yet been studied previously. In present study, we thus investigated whether EPOs could inhibit neointimal formation by inducing VSMC apoptosis in balloon-injured rat carotid artery.

## Materials and Methods

### Chemicals and reagents

EPO-B and D were synthesized as previously described [28]. PTX was purchased from Sigma-Aldrich (St. Louis, MO, USA). Other chemical sources are follows: PDGF-BB, R&D Systems (Minneapolis, MN, USA); Pifithrin-α, Calbiochem (San Diego, CA, USA); and all other chemical reagents were from Sigma-Aldrich. Cell culture materials were purchased from Gibco Life Technologies (Grand Island, NY, USA). The siRNA species for p53 was purchased from Bioneer (Dajeon, Korea). Oligofectamine was purchased from Invitrogen (Grand Island, NY, USA).

### Ethics statement and animals

This study was carried out in strict accordance with the recommendations in the Guide for the Care and Use of Laboratory Animals of the National Institutes of Health. All procedures involving experimental animals and the protocol were approved by the Committee on the Ethics of Animal Research of Chungbuk National University (Cheongju, Chungbuk, Korea) (CBNUR-792-15). Adult male Sprague Dawley (SD) rats were obtained from Charles River Laboratory (Tokyo, Japan) and housed in the Center for Experimental Animals at Chungbuk National University. Animals were maintained under conventional housing conditions at 23 ± 2°C with a controlled 12 light/dark cycle, and drinking water and rodent chow diet were provided *ad libitum* throughout the experiment.

### Rat carotid artery injury model and treatment

Rat carotid balloon injury procedures were performed as previously described [26]. Male SD rats (250–300 g) were anesthetized by intra-peritoneal injection of a mixture of xylazine (6.7 mg/kg) and ketamine (50 mg/kg). The surgical site was epilated, disinfected with Betadine, and a ventral mid-line incision was made in the neck using micro-scissors. The right carotid artery was injured by a size 2F Fogarty balloon embolectomy catheter (Baxter, McGraw Park, IL, USA) as previously described [26]. The treatment groups were divided into four groups: vehicle-control (vehicle alone), EPO-B, EPO-D, and PTX (*n* = 5/group). Immediately following balloon injury, artery was washed with PBS and 100 μL of 30% Pluronic F-127 gel solution, a thermosensitive amphiphilic polymer, containing EPO-B (20 μg/rat) or EPO-D (20 μg/rat), or PTX (100 μg/rat) was applied to the exposed adventitial surface of injured carotid artery using a 1 mL syringe with an 18 gauge blunted-tip needle. Sham surgery control animals were operated on as described above except the balloon-injury. In vehicle-control and sham-control animals, 100 μL of 30% Pluronic F-127 gel solution with vehicle (normal saline containing 10% ethanol) alone was applied to the right carotid artery. The surgical incision was then closed with 6–0 monofilament sutures and a synthetic absorbable surgical tissue adhesive (Tissuemend II SC; Veterinary Product Laboratory, Phoenix, AZ, USA), and analgesic buprenorphine (0.1 mg/kg) was administrated subcutaneously. The rats were monitored until recovery in a chamber on a heating pad. Animals were maintained for 2 weeks on the normal chow diet following surgery.

### Primary VSMC isolation and culture

VSMCs were obtained from thoracic aorta of SD rats (male, 180–200g) by a standard enzymatic digestion technique as previously described [29]. Briefly, rats were sacrificed with an overdose of pentobarbital sodium (100 mg/kg) by intra-peritoneal injection before the aorta was excised. Aortas were isolated, the viscera was dissected away, and the resulting cleaned vessel was digested with collagenase and elastase to remove adventitia and dissociate the VSMCs. Isolated cells were grown in Dulbecco's modified eagle’s medium (DMEM) supplemented with 10% fetal bovine serum (FBS), 100 IU/mL penicillin, 100 μg/mL streptomycin, 8 mM HEPES, and 2 mM L-glutamine at 37°C in a humidified incubator atmosphere of 95% air and 5% CO_2_. VSMCs from passages 4 to 7 were used for experiments.

### *In vitro* VSMC proliferation assay

Cell proliferation was assayed by using a BrdU cell proliferation assay kit (BioVision, Milpitas, CA, USA). In brief, VSMCs were seeded in 96-well culture plates at 1 × 10^4^ cells/well, and cultured DMEM containing 10% FBS at 37°C for 24 h. Cells were then serum starved for 12 h prior to the treatment with or without EPO-B or EPO-D (0.1 to 100 nM), or dimethyl sulfoxide (DMSO, final concentration of 0.1%) as a vehicle control in low-serum (0.5% FBS) medium. After 24h, the cells were stimulated by 50 ng/mL of PDGF-BB. Following incubation for 24 to 72h, BrdU incorporation assay was performed according to manufacturer’s instructions.

### *In vitro* VSMC apoptosis assay

Apoptotic cell death was determined by observing morphological changes and with the terminal deoxynucleotidyl transferase-mediated dUTP nick end labeling (TUNEL) assay as previously described [30]. Briefly, cells were seeded on an eight chambered glass culture slide (BD Biosciences, Franklin Lakes, NJ, USA) at a density of 1 × 10^4^ cells/well and cultured for 24 h, and then cells were serum starved for 12 h prior to the treatment with EPO-B or EPO-D (1 to 100 nM), PTX (100 nM), or DMSO (final concentration of 0.1%) as a vehicle control in the presence of PDGF-BB (50 ng/mL). After 48h, the cells were washed with phosphate-buffered saline (PBS), fixed with 4% paraformaldehyde, and then permeabilized with PBS containing 0.5% Tween-20. After washing with PBS, cells were processed for TUNEL staining by using *in situ* Cell Death Detection Kit (Roche Diagnostics GmbH, Mannheim, Germany) according to manufactures’ instructions. Cells were counterstained using 4′,6-diamidino-2-phenylindole (DAPI) and mounted using fluorescence mounting medium. Samples were imaged using a fluorescence microscope (200× magnification). Total number cells (DAPI positive cells) in a given area were manually counted, and apoptotic cell death was calculated as the percentage of TUNEL-positive cells out of the total number of cells.

### Mitotic catastrophe assessment

Mitotic catastrophe of VSMCs was determined by DAPI staining as previously described [11]. In brief, cells were cultured on an eight chambered glass culture slide (BD Biosciences) at a density of 1 × 10^4^ cells/well and cultured for 24 h, and then cells were serum starved for 12 h prior to the treatment with EPO-B or EPO-D (1 to 100 nM), PTX (100 nM), or DMSO (final concentration of 0.1%) as a vehicle control in the presence of PDGF-BB (50 ng/mL). After 48h, the cells were washed with PBS, fixed in 4% neutral buffered formalin, washed twice with PBS, and stained with DAPI solution. After 2 washes, slides were mounted using fluorescence mounting medium. The cells were evaluated and images were captured using a florescence microscope (200× magnification). Mitotic catastrophe was defined by abnormal morphological change of nuclear with multiple nuclei, nuclear condensation, or nuclear membrane fragmentation.

### Immunofluorescence staining on cultured VSMCs

Cells were cultured on an eight chambered glass culture slide (BD Biosciences) at a density of 1 × 10^4^ cells/well and cultured for 24 h, and then cells were serum starved for 12 h prior to the treatment with EPO-B or EPO-D (10 nM), or DMSO (final concentration of 0.1%) as a vehicle control. After 24h, the cells were stimulated with 50 ng/mL of PDGF-BB for 24h, then cells were immunofluorescently labeled with antibodies as previously described [31]. Primary antibodies used were mouse monoclonal anti-β-tubulin (Clone TUB 2.1, Sigma-Aldrich), rabbit polyclonal anti-p53 (FL-393, Santa Cruz Biotechnology, Santa Cruz, CA, USA), rabbit polyclonal anti-Bax (Δ21, Santa Cruz Biotechnology), and rabbit polyclonal anti-caspase-3 (CPP-32, Cell Signaling Technology Inc., Beverly, MA). Secondary antibodies used were Alexa Fluor 488-conjugated goat anti-mouse IgG or Alexa Fluor 568-conjugated donkey anti-rabbit IgG (Jackson ImmunoResearch Laboratories, West Grove, PA, USA). After nuclear staining with DAPI and mounting in anti-fade medium (Vector Laboratory, Burlingame, CA, USA), immunofluorescence images were acquired using a confocal laser scanning microscope (TCS SP2, Leica Microsystems AG, Wetzlar, Germany) equipped with a 63× oil immersion objective.

### Immunoblotting analysis

VSMCs were seeded into 100 mm culture dishes at a density of a density of 5 × 10^5^ cells. When cells reached up to 70% confluence, and then cells were serum starved for 12 h prior to the treatment with EPO-B or EPO-B (1 to 100 nM), or DMSO in low-serum medium. After 24h, the cells were stimulated with 50 ng/mL of PDGF-BB for 24h, then the whole cell lysates were obtained and the proteins were separated on 10% to 15% SDS-PAGE. The proteins were transferred to PVDF membrane, and membranes were blocked with 5% skim milk. Immunoblotting analysis was performed as previously described [30, 31]. Briefly, The protein transfer membrane were proved with 1:500 to 1:2000 dilution of antibodies against PCNA, p21, total-p53, phosphor-p53 (Ser15), Bax, and cleaved-caspase-3 from Cell Signaling Technology (Danvers, MA, USA) in 5% skim milk over night at 4°C, followed by incubation with alkaline phosphatase-conjugated secondary antibodies. The β-actin was used as a loading control. Protein expression was visualized by a chemiluminescence reagent (Millipore, Billerica, MA, USA), and detected by using a digital chemiluminescence imaging system equipped a charge coupled device (CCD) camera (Fusion-FX, Fisher BioTech Ltd., Wembley, Australia).

### Aorta tissue preparation and immunohistochemical staining

Rats were sacrificed by CO_2_ inhalation and pressure perfused with saline containing heparin, and arteries were harvested and fixed in 10% buffered formalin, and embedded in paraffin to prepare cross-sections for quantitative morphometry analysis and immunohistochemical staining. Carotid artery cross sections (5 μm thickness) were mounted on microscope slides and the expression of proliferation cell nuclear antigen (PCNA), p53 and active-caspase-3 in tissue sections was evaluated by immunohistochemical stains using primary anti-PCNA, p53 or active-caspase-3 antibodies, and secondary biotinylated antibodies as previously described [31]. Micrographs were taken with a light microscope (Olympus IX 71 with DP71 camera, Japan) at 10× or 20× magnification.

### Detection of *in situ* apoptosis on aortic tissues

Carotid artery cross sections were fixed with 4% paraformaldehyde, and then permeabilized with PBS containing 0.5% Tween-20. After washing with PBS, tissues were processed for TUNEL staining by using *in situ* Cell Death Detection Kit (Roche Diagnostics GmbH) according to manufactures’ instructions. Tissues were mounted using fluorescence mounting medium, and were imaged using a fluorescence microscope (20× magnification). The total cell number in a given area was manually counted based on DAPI nuclear staining. The percentage of apoptotic cells (TUNEL-positive cells) in arterial wall and neointima was calculated as described above.

### Statistical analysis

Statistical analysis was performed using Graph-Pad Prism 5.0 software (GraphPad Software Inc., La Jolla, CA, USA). Experimental results are expressed as means±SEM. One-way analysis of variance (ANOVA) was used for multiple comparisons followed by Dunnett’s test. Differences with *P* values of <0.05 were considered statistically significant.

## Results

### Epothilones inhibit proliferation and induce apoptosis in VSMC

**Fig 1:**
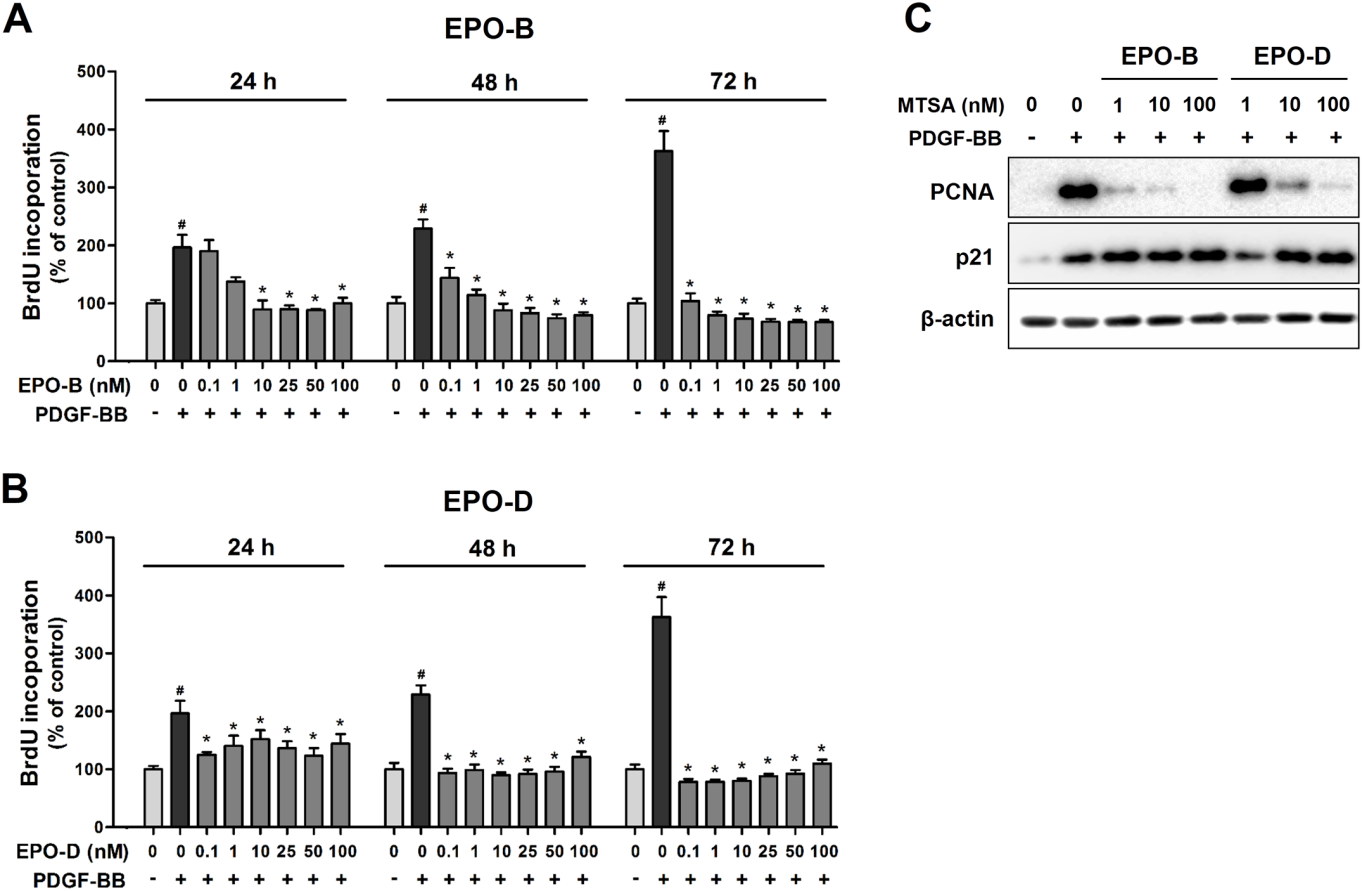
EPO-B and EPO-D inhibits VSMCs proliferation. **(A-B)** VSMCs were cultured in 96-well culture plate for 24 h, then serum starved for 12h. Cells were treated with 0.1 to 100 nM of EPO-B (**A**) and EPO-D (**B**) or 0.1% DMSO (vehicle) for 24h, cells were stimulated with PDGF-BB (50 ng/mL), and cell proliferation was then determined by BrdU incorporation assay (*n* = 8 each) at 24, 48, and 72 h intervals. Data shown as mean ± SEM. ^**#**^*P*<0.05 indicate significantly different from serum-free/DMSO-treated control and *P<0.05 indicate significantly different from PDGF-BB/DMSO-treated control. (**C**) VSMCs were treated with EPO-B and EPO-D (1 to 100 nM) or 0.1% DMSO in serum-free media for 24h, then stimulated with PDGF-BB (50 ng/mL). After 24 h, cells were lysed and PCNA and p21 expression was analyzed by Western blot.

Initially, the effect of EPOs on rat VSMC proliferation was evaluated. A time course study showed that treatment of EPO-B (Fig 1A) and EPO-D (Fig 1B) for 24 h, effectively inhibited the PDGF-BB-induced VSMC proliferation in a dose-dependent manner, consistent with previous studies [26, 27]. In further, we found that the inhibitory effect of EPO-B and EPO-D on VSMC proliferation lasted at least 72 h after the treatment, and its inhibition rates were enhanced by increasing treatment time (S1 Table). In addition, treatment of EPOs reduced cell density in a dose-dependent manner (S1 Fig). These results indicated that EPO-B and EPO-D has long-lasting inhibitory effects on the proliferation and growth of VSMCs. We further found that treatment of both EPO-B and EPO-D effectively downregulated PDGF-BB-induced PCNA expression, while upregulated p21 expression in VSMCs (Fig 1C), suggesting that EPOs inhibit PDGF-BB-induced VSMC proliferation and growth by regulating cell proliferation and cell cycle regulatory protein expression.

**Fig 2:**
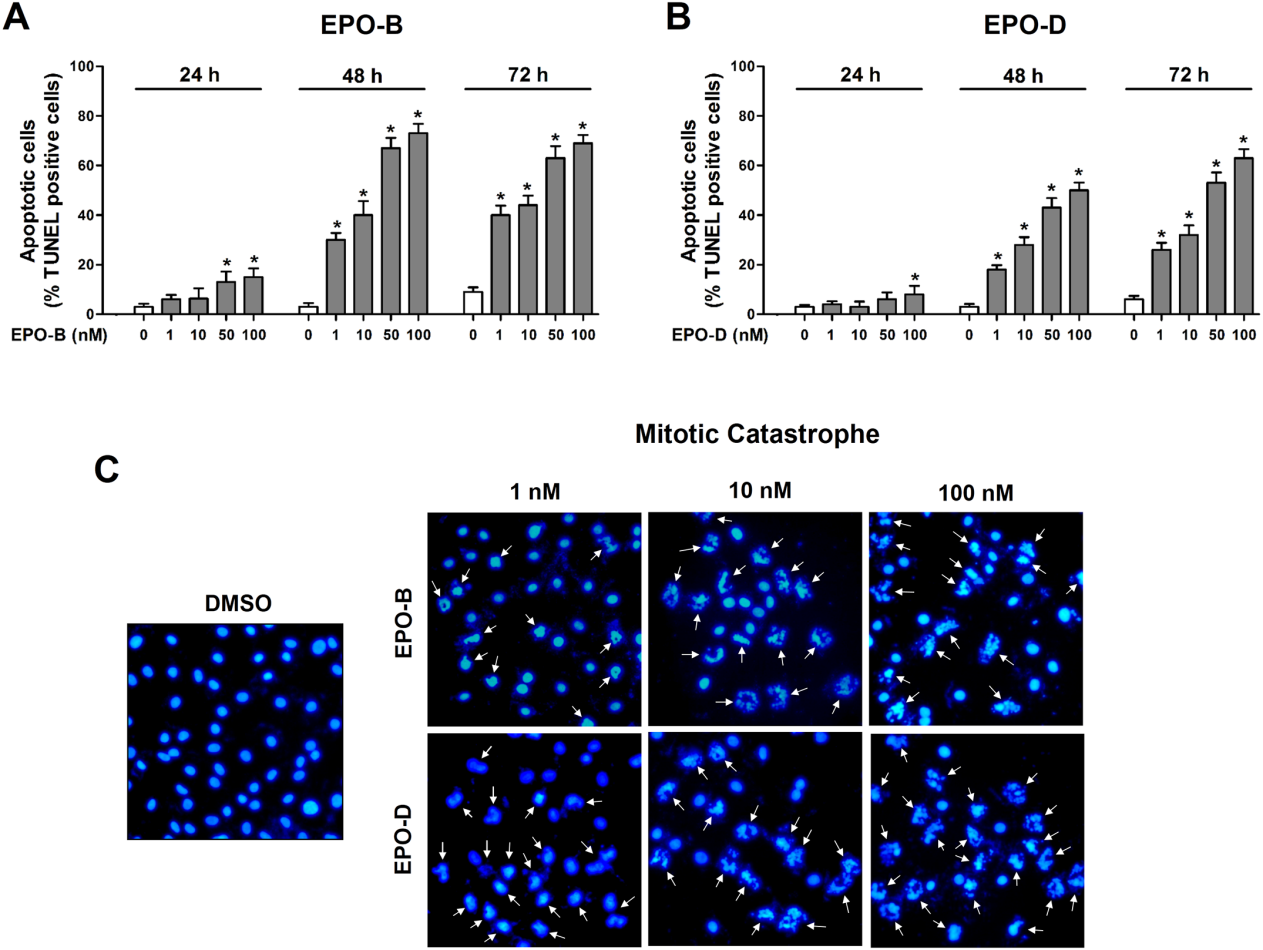
EPO-B and EPO-D induces apoptotic cell death and mitotic catastrophe in VSMCs. **(A-B)** VSMCs were cultured in an eight chambered glass culture slide, and then serum starved for 12h, cells were then treated with 1 to 100 nM of (**A**) EPO-B and (**B**) EPO-D or 0.1% DMSO (vehicle) for 24h, cells were stimulated with PDGF-BB (50 ng/mL). After 24h, cell apoptosis was determined by TUNEL-assay. The apoptotic index was determined as the TUNEL-positive cell number divided by the total cell number (*n* = 3 each). Data are shown as mean ± SEM. *P<0.05 indicate significantly different from DMSO-treated control cells. (**C**) Cell mitotic catastrophe were determined by nucleus DAPI-staining (blue) at 24 h post-PDGF-BB-stimulation. Mitotic catastrophe was characterized by cytoplasmic blebbing, condensation and irregularities in shape under fluorescence microscopy (magnification, 40×). The arrows indicated the mitotic catastrophe changed cells. Representative images of each experimental group are shown.

Next, we investigated whether treatment of EPOs induces cell death in proliferating VSMCs by using *in situ* TUNEL-assay, which detect nuclear DNA fragmentation (apoptotic cell death), and DAPI nuclear staining, which revealed mitotic catastrophe. We found that treatment of both EPO-B and EPO-D significant and dose-dependently increased the proportion of apoptotic cells (TUNEL-positive) from 48 h post-treatment, compared with vehicle-treated control (Fig 2A and 2B, S2 Fig). Further, cell morphological changes (S3 Fig), giant micronucleated and mitotic catastrophe (Fig 2C) were observed both in EPO-B and EPO-D treated VSMCs. In addition, we confirmed that PTX, a positive control MTSA, strongly induces apoptosis, cell morphological changes and mitotic catastrophe (S2 and S3 Figs). Taken together, our results suggested that both EPO-B and EPO-B may inhibit PDGF-BB-induced cell growth and proliferation of VSMCs through increasing of apoptotic cell death as well as regulating proliferation and cell cycle regulatory protein expression.

### Activation of p53 is involved in the pro-apoptotic effect of epothilones in VSMCs

**Fig 3:**
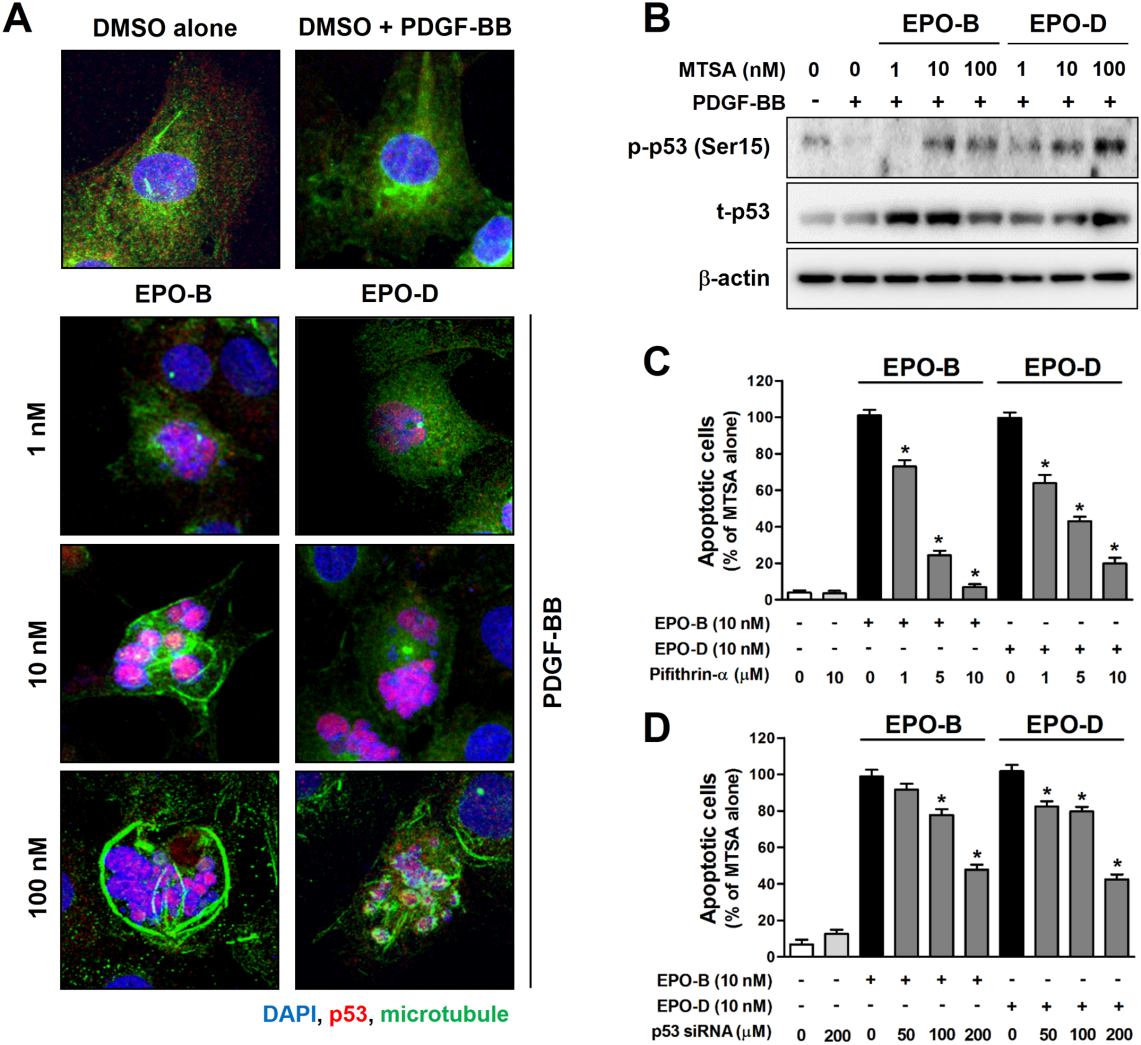
EPO-B and EPO-D induces apoptosis through p53 activation in VSMCs. (**A**) VSMCs were cultured in an eight chambered glass culture slide, and then serum starved for 12h. After treatment of EPO-B and EPO-D (1 to 100 nM), or 0.1% DMSO (vehicle) for 24h, cells were stimulated with PDGF-BB (50 ng/mL) for 24 h. Activation of p53 and microtubule polymerization were determined by immunofluorescence staining with p53 (red) and β-tubulin (green) antibodies. Nuclei were stained with DAPI (blue). Images shown are representative confocal-laser-scanning microscope (magnification, 63×). (**B**) VSMCs were treated with EPO-B and EPO-D (1 to 100 nM), or 0.1% DMSO in serum-free media for 24h, then stimulated with PDGF-BB (50 ng/mL). After 24 h, cells were lysed and phosphorylated p53 (p-p53, Ser15) and total p53 (t-p53) expression was analyzed by Western blot. To clarify the involvement of p53 on EPOs-induced apoptosis, cells were treated with EPO-B and EPO-D (10 nM) with/without 1–10 μM pifithrin-α, a p53 inhibitor, (**C**) or p53 siRNA (**D**) for 24 h, and were then stimulated with 50 ng/ml PDGF-BB for 24 h. The percent of VSMCs apoptosis was measured by TUNEL-assay. Data are shown as mean ± SEM (*n* = 3). *P<0.05 indicate significantly different from EPOs-alone treated control cells.

To determine the underlying pro-apoptotic mechanism of EPOs in VSMCs, we examined whether EPOs induce apoptotic signaling pathway in VSMCs. Since p53 has been implicated in a growing number of biological processes, including cell growth, cell cycle arrest and apoptosis [32], we investigated whether EPOs regulate p53 activation associate with microtubule dynamics in PDGF-BB-stimulated VSMCs. We firstly confirmed that that EPO-B and EPO-D strongly induced microtubule polymerization in a dose- and time-dependent manner (S4 Fig). As shown in Fig 3A, we found that cellular expression of p53 was strongly increased both in EPO-B- and EPO-D-treated VSMCs. Interestingly, we observed that treatment with 1 nM of EPO-B or EPO-D resulted in enhanced p53 nuclear accumulation with suppressed microtubule dynamics, but without apparent effects on microtubule polymerization in PDGF-BB-stimulated VSMCs. We further found that treatment with higher concentration (over 10 nM) of EPO-B and EPO-D resulted in predominant p53 accumulation in micronucleated cells with extensive microtubule polymerization (Fig 3A). In agreement with the immunofluorescence staining results, treatment of EPO-B and EPO-D increased phosphorylation of p53 as well as total p53 expression in PDGF-BB-stimulated VSMCs (Fig 3B).

Next, we tested whether EPOs-mediated p53 activation is involved in EPOs-induced apoptotic cell death in proliferating VSMCs by using pifithrin-α, a reversible inhibitor of p53-meidated apoptosis and p53-dependent gene transcription [33], and p53 siRNA. We found that the pretreatment with pifithrin-α and p53 siRNA significantly rescued apoptotic cell death in both EPO-B and EPO-D-treated VSMCs in a dose-dependent manner (Fig 3C and 3D). These results suggested that pro-apoptotic effect of EPOs in VSMCs might be due to activation of p53-dependent apoptotic signaling pathway.

### Epothilones induce p-53-dependent apoptotic signaling pathway

**Fig 4:**
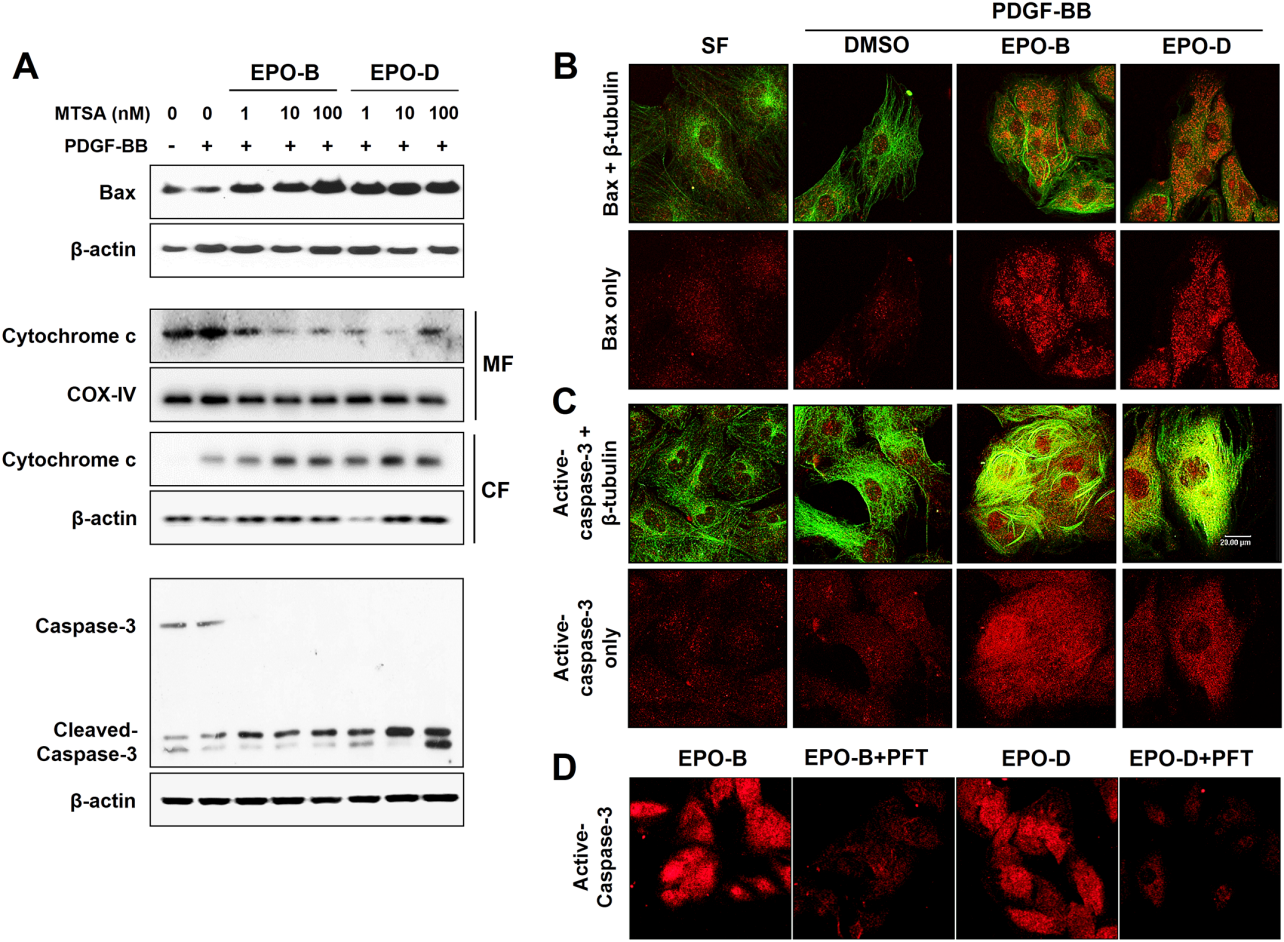
EPO-B and EPO-D induces p-53 dependent apoptotic signaling pathway. (**A**) VSMCs were treated with EPO-B and EPO-D (1 to 100 nM), or 0.1% DMSO in serum-free media for 24h, then stimulated with PDGF-BB (50 ng/mL). After 24 h, cells were lysed and Bax, mitochondrial cytochrome c release and cleaved caspase-3 expressions were analyzed by Western blot. MF: mitochondrial fraction, CF: cytosolic fraction. (**B-C**) VSMCs were cultured in an eight chambered glass culture slide, and then serum starved for 12h. After treatment of EPO-B and EPO-D (10 nM), or 0.1% DMSO (vehicle) for 24h, cells were stimulated with PDGF-BB (50 ng/mL) for 24 h. For immunofluorescence staining, cells were processed for double immunofluorescence labeling with antibodies against (**B**) Bax (red) or (**C**) cleaved-caspase-3 (red), and β-tubulin (green). Images were acquired using a confocal-laser-scanning microscope and the fluorescent micrographs (magnification, 63×). (**D**) Cells were starved for 12 h prior to the treatment with EPO-B or EPO-B (10 nM) with/without 10 μM pifithrin-α (p53 inhibitor). After 24h, the cells were stimulated with 50 ng/mL of PDGF-BB for 24h, and processed for single immunofluorescence labeling (active-caspase-3, red).

To further investigate the underlying pathway of EPOs-induced apoptosis, we evaluated the expression of well-known p53-dependent apoptotic proteins such as Bax, cytochrome c and caspase-3 by Western blot analysis. Treatment with both EPO-B and EPO-D strongly increased the expression of Bax in PDGF-BB-stimulated VSMCs in a dose-dependent manner (Fig 4A upper panels). Cytochrome c, a component of the mitochondrial electron transfer chain, is released into the cytosol during the apoptotic cell death. Therefore, the accumulation of mitochondrial cytochrome c release was determined in EPOs-treated VSMCs. We found that treatment VSMCs with EPOs increased the expression of cytochrome c in cytoplasm and reduced its expression in mitochondria (Fig 4A middle panels). In order to test whether EPOs induces caspase-dependent apoptotic pathway, the expression of cleaved-caspase-3 was determined. Results showed that treatment with EPOs deceased the level of procaspase-3 and upregulated the expression of cleaved caspase-3 in PDGF-BB-stimulated VSMCs (Fig 4A lower panels).

In agreement with the western blot analysis, *in situ* immunofluorescence confocal laser scanning microscopy images showed that treatment with EPOs enhanced cellular expression of Bax and active-caspase-3 with extensive microtubule polymerization (Fig 4B and 4C). Interestingly, we further found that EPOs-induced active-caspase-3 expression was strongly suppressed by pifithrin-α treatment (Fig 4D). Taken together, our findings suggested that EPOs induces apoptosis by activating the p53-dependent apoptotic signaling pathway (Bax/cytochrome c/casepase-3).

### Epothilones inhibit neointimal formation and induce apoptosis in the balloon-injured rat carotid artery

**Fig 5:**
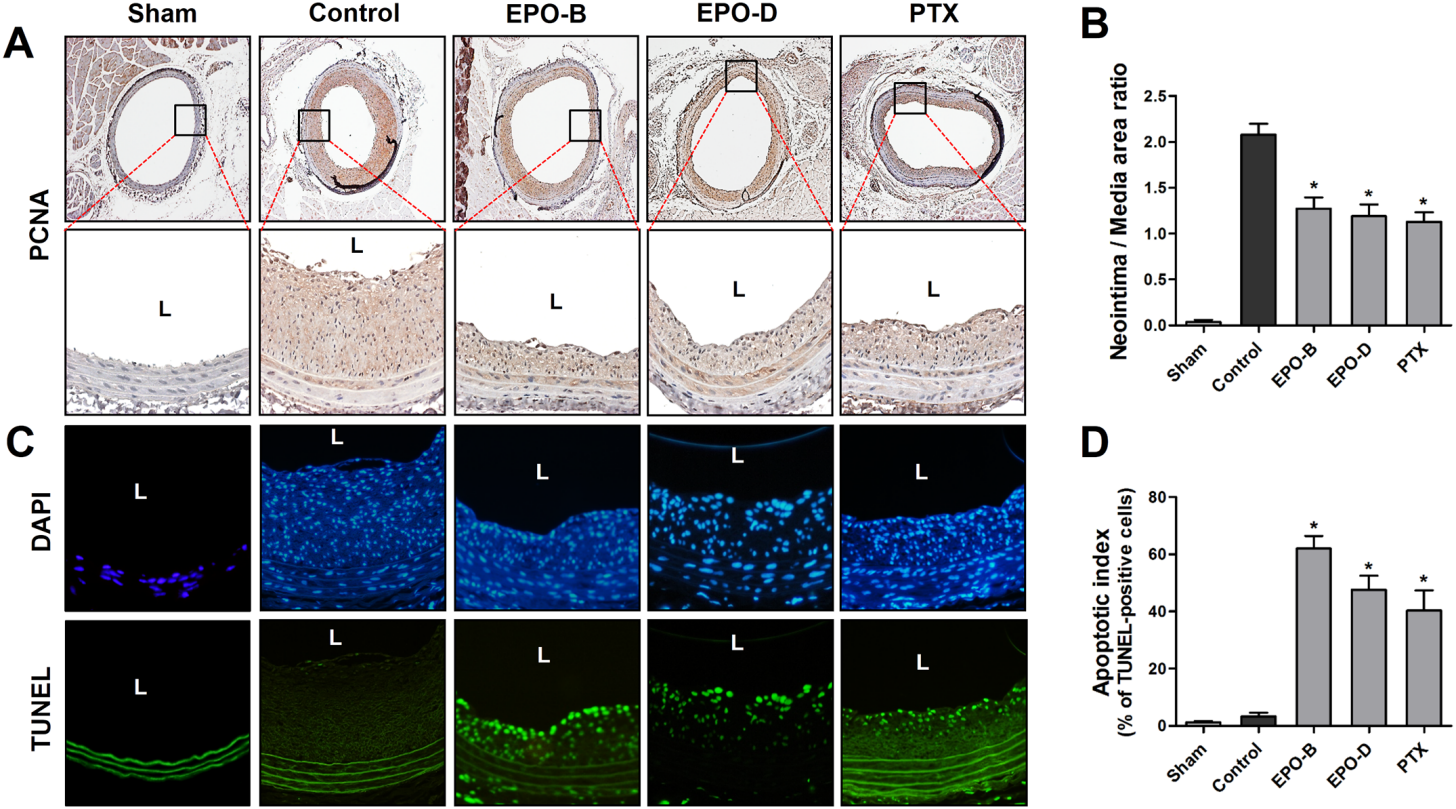
Treatment of EPO-B and EPO-D inhibits neointimal hyperplasia and induces apoptosis in the regions of neointimal hyperplasia. (**A**) Carotid sections of each indicated groups were stained with PCNA antibody. Images shown are representative microscopy images (*n* = 5 each; magnification, 4×). The lower panels show magnified views of neointimal hyperplasia regions. Nuclei (blue) and protein expression (brown) are shown. L = lumen of the artery. (**B**) Neointima/Medial area ratio was quantified. Data shown as mean ± SEM. *P<0.05 indicate significantly different from injured-control group. (**C**) Apoptotic cell death in carotid tissues (*n* = 5) was determined by *in situ* TUNEL-assay (green). Images shown are representative fluorescence microscopy images (magnification, 20×). Blue: DAPI and lined-green: autofluorescent elastic lamina. (**D**) The apoptotic index was determined as the TUNEL-positive cell number (lower panels) divided by the total cell number (DAPI-stained cells, upper panels). Data are shown as mean ± SEM (*n* = 5). *P<0.05 indicate significantly different from balloon-injured control group.

To validate whether our *in vitro* findings were also pathophysiologically relevant *in vivo*, we tested effects of EPOs on neointimal formation in a rat carotid balloon-injury model. Rat carotid arteries were subjected to balloon injury and locally treated with EPO-B (20 μg/rat) or EPO-D (20 μg/rat) or PTX (100 μg/rat), a positive control MTSA, using Pluronic F127 gel as described above in Methods. In our experimental condition, balloon injury-induced neointimal hyperplasia was evident in vehicle-treated injured-control animals at 14 days post surgery, but not in sham-control group (Fig 5A). We found that local delivery of EPO-B and EPO-D significantly inhibits balloon-injury induced neointimal lesion formation compared to vehicle control group with 30.24% and 34.14% reduction, respectively, in the intima/media area ratio, which was consist with previous findings [26, 27]. In addition, we confirmed that neointimal formation was significantly reduced by treatment with PTX (Fig 5A and 5B). We further found that injury-induced neointimal lesion formation in the carotids of control rat showed correlation with increased cell proliferation (PCNA expression) that was markedly decreased in EPO-B-, EPO-D- and PTX-treated carotids (Fig 5A).

**Fig 6:**
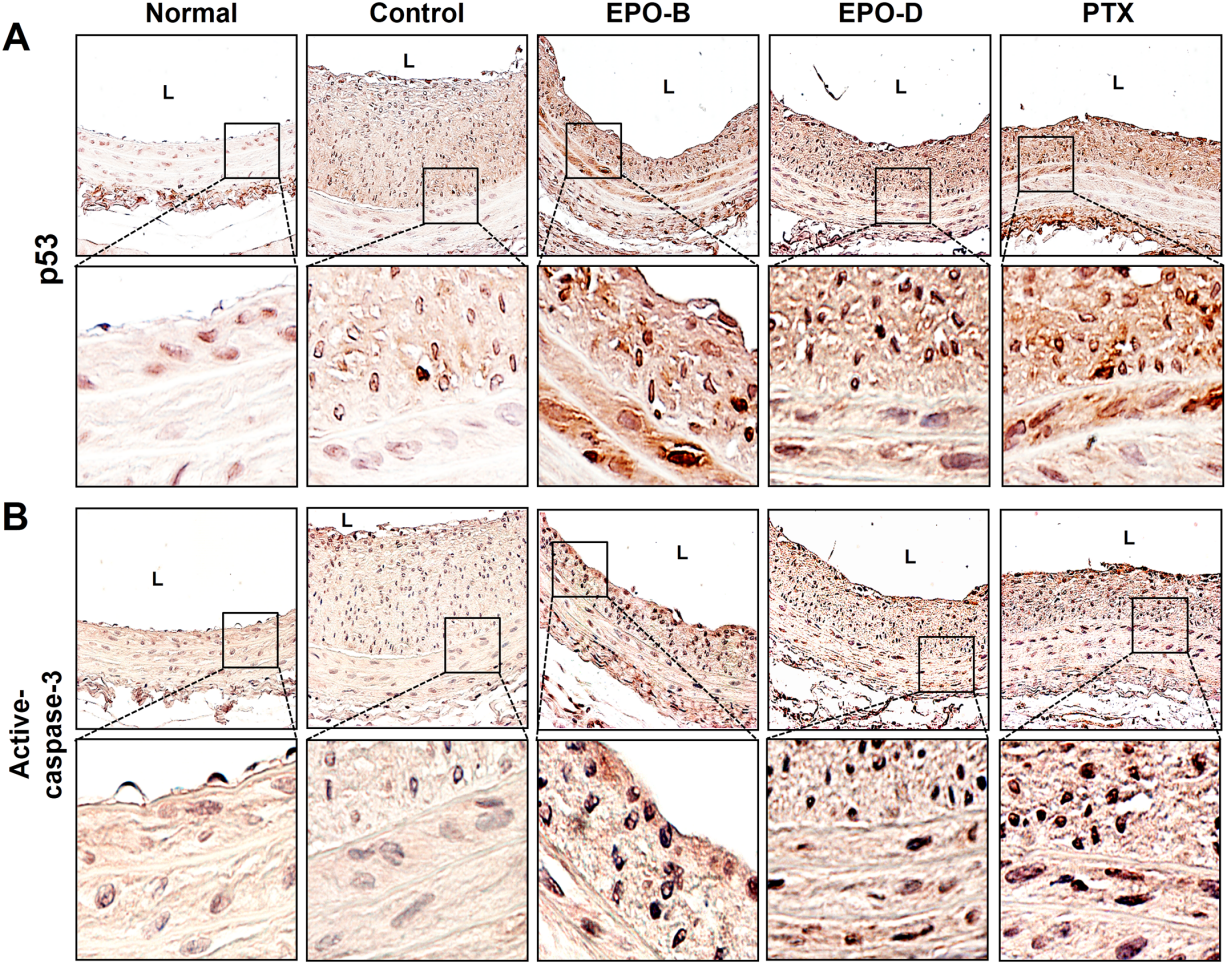
Treatment of EPO-B and EPO-D increase the expressions of p53 and active-caspase-3 in the regions of neointimal hyperplasia. Carotid sections of each indicated groups were stained with p53 (**A**) or active-caspase-3 (**B**) antibodies. Images shown are representative microscopy images (*n* = 5; magnification, 20×). The lower panels show magnified views of neointimal-hyperplasia regions (magnification, 40×). Nuclei (blue) and protein expression (brown) are shown. L = lumen of the artery.

Next, we investigated whether the inhibitory effect of EPOs on neointimal hyperplasia was due to induction of apoptosis in neointimal lesions by *in situ* TUNEL-assay. As shown in Fig 5C, apoptotic cell death (TUNEL-positive) was not detected in sham control and vehicle control group. In comparison, EPO-B- and EPO-D-treated carotids showed significantly induced apoptosis within the region of neointimal hyperplasia with apoptotic index values (calculated as the percentage of TUNEL-positive cells versus total nucleated cells in given area) of 61.97% and 47.55%, respectively (Fig 5C). Similar level of apoptosis was detected in the PTX-treated group (40.26%). These results demonstrated that treatment of EPOs might reduce the balloon-injury-induced neointimal lesion formation by inducing apoptosis. In addition, we confirmed that the expression of p53 and active-caspases-3 was increased in neointimal lesion of both EPOs and PTX-treated group compared with vehicle-treated control (Fig 6A and 6B).

These *in vivo* results in a rat balloon-injury model, taken together with the *in vitro* findings shown above, provide strong evidence that EPO-B and EPO-D inhibits neointimal hyperplasia by inducing VSMC apoptosis through the p53-dependent apoptotic signaling pathway.

## Discussion

The central hypothesis of the present study was that EPOs induce apoptosis in hyper-proliferated VSMCs, thereby inhibiting neointimal hyperplasia. Here, we showed that treatment of both EPO-B and EPO-D significantly induce apoptotic cell death and mitotic catastrophe in PDGF-BB-stimulated VSMCs results in the inhibition of cell proliferation and growth. We further demonstrated that the local treatment of EPOs potently suppressed neointimal formation and induced apoptosis on neointimal region in a rat balloon-injury model. In addition, we found that p53-dependent apoptotic signaling pathway plays a critical role in the inhibitory effect of EPOs on neointimal lesion formation.

The marked abnormal accumulation of cells within the intimal space by alterations in homeostatic balance between cell growth and cell death plays a key role in the process of neointimal hyperplasia [3, 34]. Uncontrolled neointimal tissue accumulation of VSMCs shows some similarity with the tumor cell growth and benign tissue proliferation [35]. Thus, the regulation of apoptosis has attracted considerable attention as an effective way to eliminate hyper-proliferative VSMCs in neointimal formation [11–13, 36–38]. While investigating the pro-apoptotic effect of EPOs, we initially observed that EPO-B and EPO-D potently inhibited PDGF-BB-induced VSMC proliferation in a dose- and time-dependent manner, consistent with previous studies [26, 27]. We further found that treatment of proliferating VSMCs with EPO-B and EPO-D for over 48 h significantly induce apoptosis along with cell morphological changes and mitotic catastrophe.

The tumor suppressor protein p53 is known to be involved in VSMC growth and cell death as well as restenosis and atherosclerosis development [30, 39, 40]. It has been previously reported that an increase in the nuclear translocation of p53 in cancer cells occurs in response to treatment with MTSAs [41, 42]. It is generally accepted that the MTSA-induced microtubule stabilization is the primary mechanism responsible for the increase in the p53 association with microtubules and its nuclear export [41, 43, 44] which associate with p53-dependent apoptotic pathway [41, 45–48]. In this study, we found that EPO-B and EPO-D strongly increased phosphorylation and nuclear accumulation of p53 in PDGF-BB-stimulated VSMCs. These suggested that the association between p53 and microtubule stabilization in EPOs-treated VSMCs may be linked to EPOs-induced apoptosis. In support of this hypothesis, we showed that pretreatment with a p53 inhibitor (pifithrin-α) and p53 siRNA significantly reversed the pro-apoptotic activity of EPOs in VSMCs, indicating EPOs-induced VSMC apoptosis might be mediated through the activation of p53 and its downstream apoptotic signaling pathway.

It is well documented that cell death induced through the p53 pathway is executed by the caspase proteinases, which by cleaving their substrates, lead to the characteristic apoptotic phenotype [49]. The death effector Bax is a transcriptional target of p53 and plays an essential role on the p53-dependent apoptosis, which triggers mitochondrial cytochrome c release and caspase signaling activation [49–52]. Thus, we investigated the involvement of the Bax, cytochrome c release and caspase-3 in EPOs-induced VSMC apoptosis. We found that treatment with EPOs strongly increased expression of Bax and cleaved caspase-3 as well as mitochondrial cytochrome c release into cytosol in PDGF-BB-stimulated VSMCs. Interestingly, we further found that pre-treatment of p53 inhibitor attenuated the EPOs-induced caspase-3 activation. These results indicated that pro-apoptotic effect of EPOs is mediated through the activation of p53-dependent apoptotic signaling pathway.

Since we have showed pro-apoptotic effects of EPOs in proliferating VSMCs *in vitro*, we investigated the possible involvement of EPOs-mediated apoptosis on neointimal formation in an *in vivo* setting. We employed rat carotid balloon-injury model, a widely used model of neointimal hyperplasia and restenosis, to test anti-neointimal hyperplasia property of EPOs by inducing VSMC apoptosis. We showed that balloon-injury-induced neointimal lesion formation was potently inhibited by local treatment with EPOs in rat carotids along with reduced cell proliferation. Moreover, our *in situ* apoptosis assay results showed that significantly induced apoptosis both in EPO-B-and EPO-D-treated carotids compared with vehicle-treated control group. Moreover, up-regulated expressions of p53 and activated-caspase-3 were found in EPOs-treated neointimal region compared with vehicle-treated group. These results indicated that p53 may be importantly associated with EPOs-induced activation of caspase-3 and Bax apoptotic pathway in VSMCs. These findings demonstrated that EPO-B and EPO-D inhibits neointimal lesion formation by inducing apoptosis in the rat carotid injury model.

In conclusion, we found that treatment of VSMC with EPOs inhibit neointimal hyperplasia in rat carotid artery by inducing VSMC apoptosis through activation of p53-dependent apoptotic signaling pathway (Bax/cytochrome c/capsepase-3). Our findings thereby provide new mechanistic insight for the potent anti-neointimal formation property of EPOs in a rodent model of neointimal hyperplasia. Therefore, knowledge gained from the current study could be translatable to humans, providing a potential clinical relevance.

## Supporting Information

S1 Fig Effect of EPO-B and EPO-D on VSMC density. After treatment of EPO-B and EPO-D (0.1 to 100 nM) or 0.1% DMSO (vehicle) for 24 h, its effect on cell density in PDGF-BB-stimulated VSMCs were determined. Representative bright field microscopic images of each experimental group are shown (*n* = 5 each).
(TIF)

S2 Fig Effect of EPO-B and EPO-D on VSMC apoptosis. (**A**) Effect of EPO-B and EPO-D on cell apoptosis in PDGF-BB-stimulated VSMCs were determined by TUNEL-assay as described in Materials and Method. Representative fluorescence microscopy images showed apoptosis induction (TUNEL-positive, green color) in 10 nM EPO-B- and 10 nM EPO-D-treated VSMCs at 24h post treatment of PDGF-BB (50 ng/mL). PTX (100 nM) was used as a positive control. (**B**) Treatment of PTX (100 nM) induces mitotic catastrophe in VSMCs at 24 h post-PDGF-BB-stimulation.
(TIF)

S3 Fig Effect of EPO-B and EPO-D on VSMC morphological change. After treatment of EPO-B (0.1 to 100 nM) or EPO-D (0.1 to 100 nM) for 24 h, its effect on cell morphological changes in PDGF-BB-stimulated VSMCs were determined. Representative bright field microscopy images of each experimental group are shown.
(TIF)

S4 Fig Effect of EPO-B and EPO-D on microtubule polymerization in VSMCs. After treatment with EPO-B (1 to 100 nM) or EPO-D (1 to 100 nM), the microtubules were stained with mouse anti-β-tubulin antibody (green) and DAPI (blue) at indicated time point. Representative confocal laser scanning microscopy images of each experimental group are shown.
(TIF)

S1 Table Inhibition rate of EPO-B and EPO-D on PDGF-BB-induced VSMC proliferation. (DOCX)
