# Supplementary figures and images for: Epothilones Suppress Neointimal Thickening in the Rat Carotid Balloon-Injury Model by Inducing Vascular Smooth Muscle Cell Apoptosis through p53-Dependent Signaling Pathway
Source: PLoS One. 2016 May 24;11(5):e0155859. doi: 10.1371/journal.pone.0155859 (PMC4878802; doi:10.1371/journal.pone.0155859)

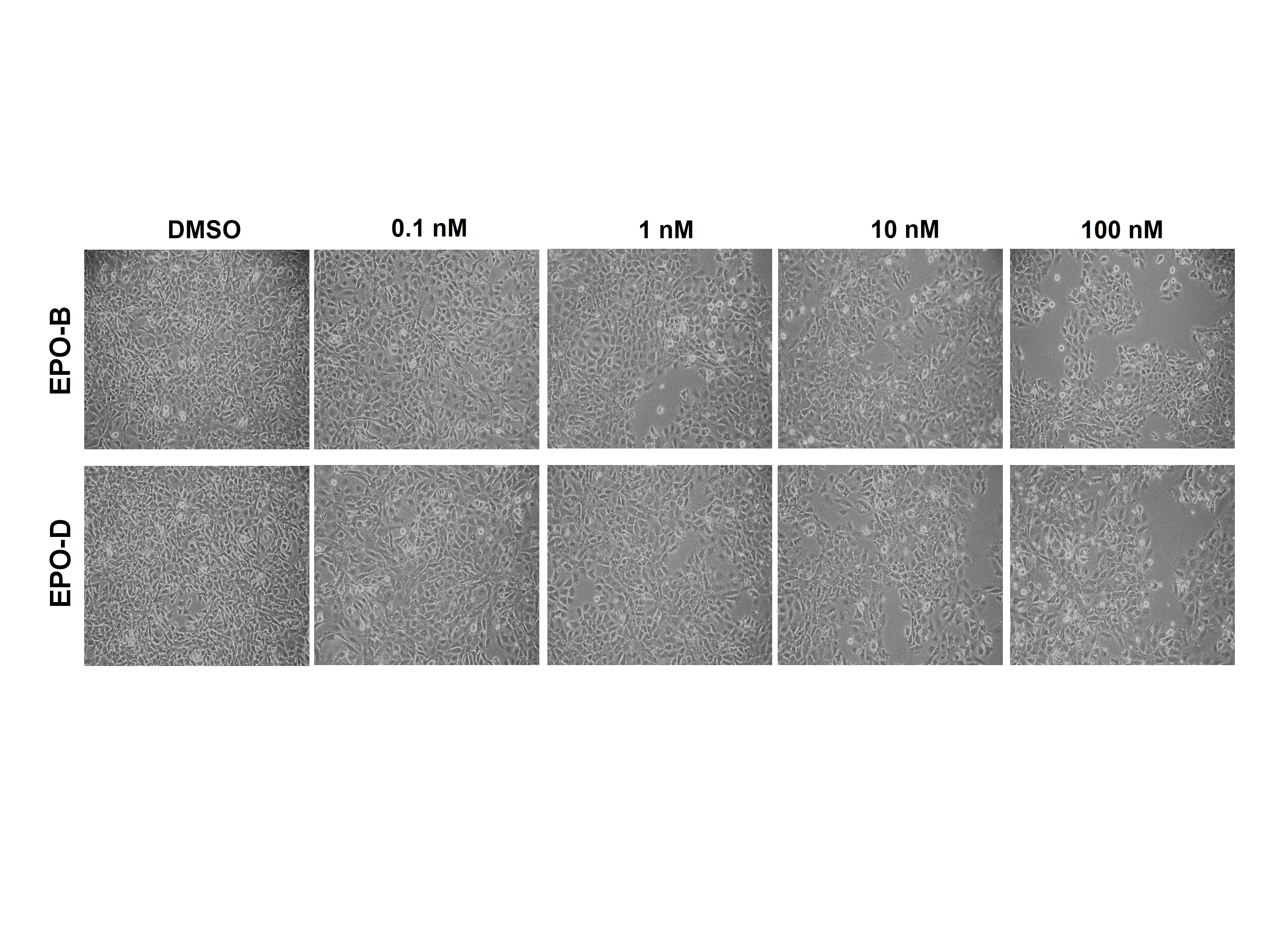

Supplement: S1 Fig — After treatment of EPO-B and EPO-D (0.1 to 100 nM) or 0.1% DMSO (vehicle) for 24 h, its effect on cell density in PDGF-BB-stimulated VSMCs were determined. Representative bright field microscopic images of each experimental group are shown (n = 5 each). (TIF) [file pone.0155859.s001.tif]

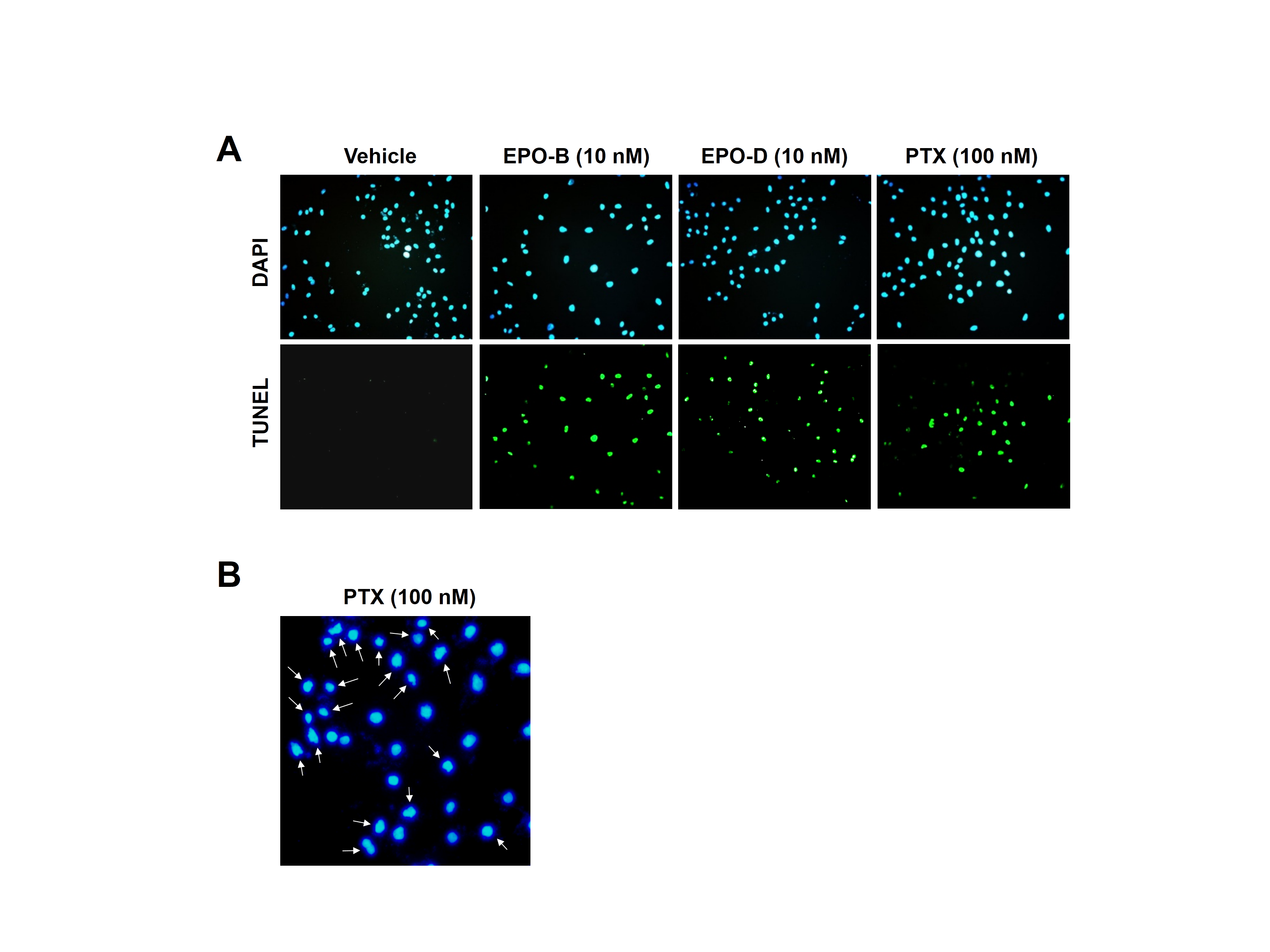

Supplement: S2 Fig — (A) Effect of EPO-B and EPO-D on cell apoptosis in PDGF-BB-stimulated VSMCs were determined by TUNEL-assay as described in Materials and Method. Representative fluorescence microscopy images showed apoptosis induction (TUNEL-positive, green color) in 10 nM EPO-B- and 10 nM EPO-D-treated VSMCs at 24h post treatment of PDGF-BB (50 ng/mL). PTX (100 nM) was used as a positive control. (B) Treatment of PTX (100 nM) induces mitotic catastrophe in VSMCs at 24 h post-PDGF-BB-stimulation. (TIF) [file pone.0155859.s002.tif]

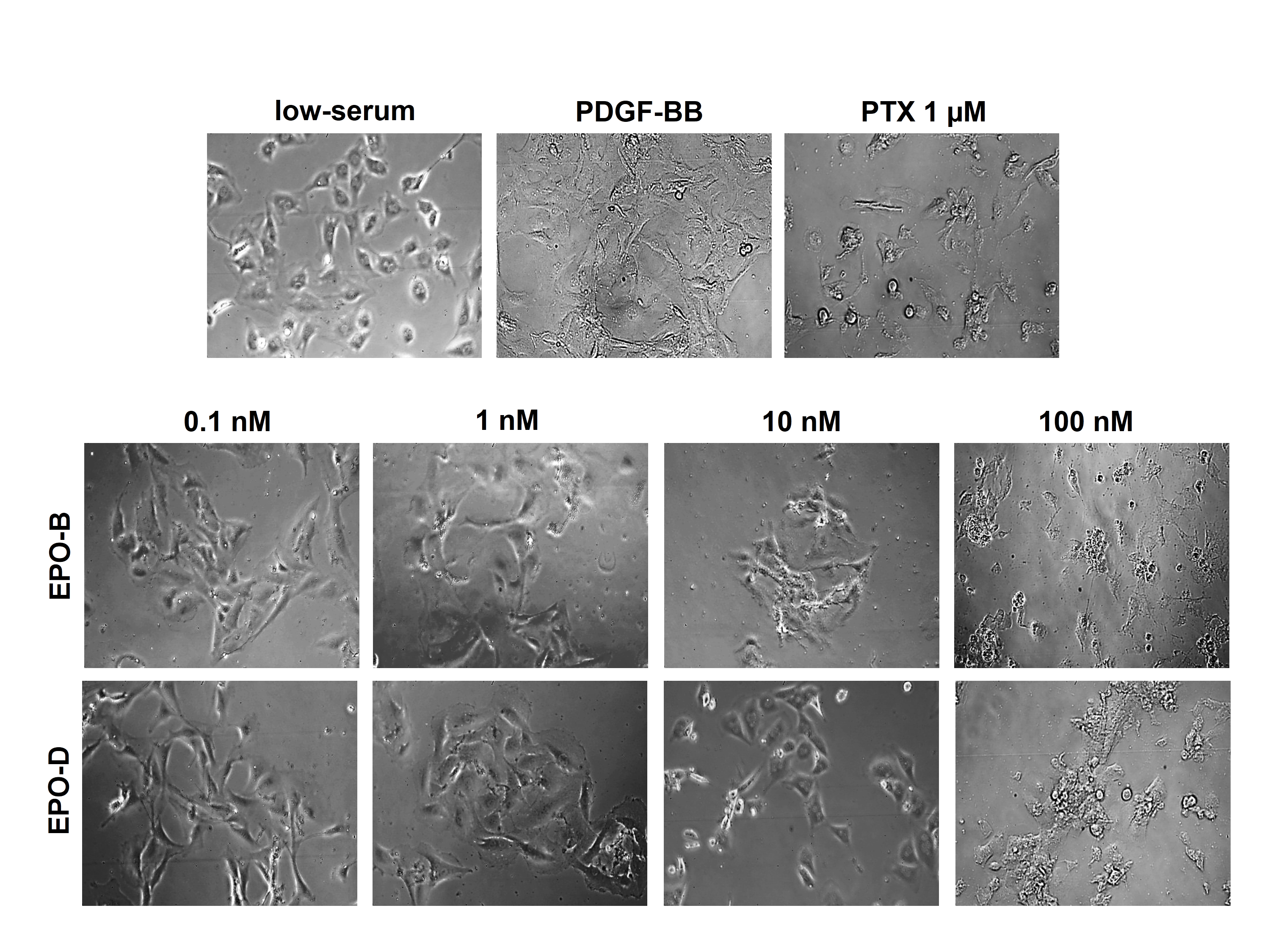

Supplement: S3 Fig — After treatment of EPO-B (0.1 to 100 nM) or EPO-D (0.1 to 100 nM) for 24 h, its effect on cell morphological changes in PDGF-BB-stimulated VSMCs were determined. Representative bright field microscopy images of each experimental group are shown. (TIF) [file pone.0155859.s003.tif]

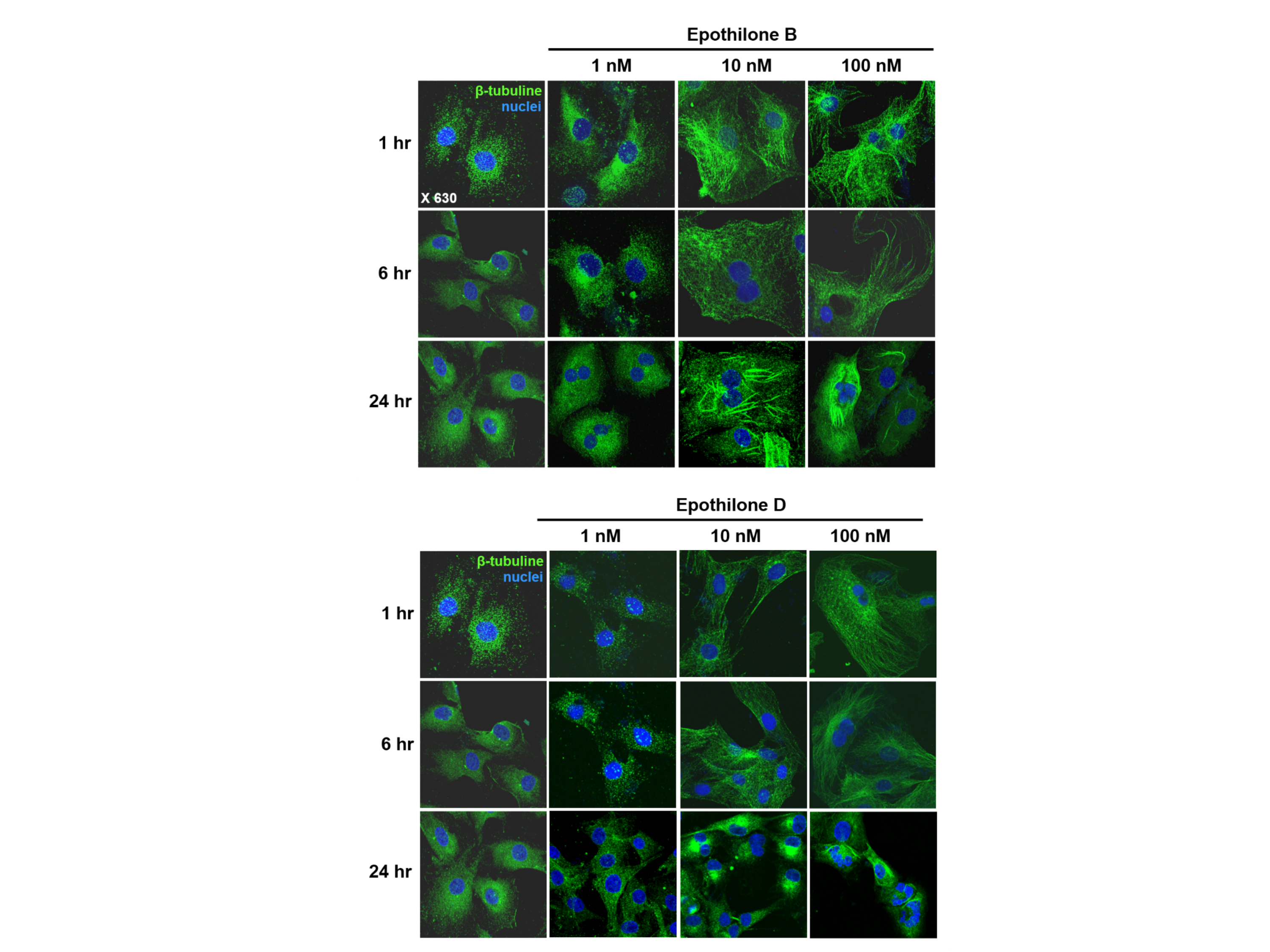

Supplement: S4 Fig — After treatment with EPO-B (1 to 100 nM) or EPO-D (1 to 100 nM), the microtubules were stained with mouse anti-β-tubulin antibody (green) and DAPI (blue) at indicated time point. Representative confocal laser scanning microscopy images of each experimental group are shown. (TIF) [file pone.0155859.s004.tif]
