# Supplementary material for: Epothilones Suppress Neointimal Thickening in the Rat Carotid Balloon-Injury Model by Inducing Vascular Smooth Muscle Cell Apoptosis through p53-Dependent Signaling Pathway
Source: PLoS One. 2016 May 24;11(5):e0155859. doi: 10.1371/journal.pone.0155859 (PMC4878802; doi:10.1371/journal.pone.0155859)
Supplement: S1 Table — (DOCX) [file pone.0155859.s005.docx]

**S1 Table. Inhibition rate of EPO-B and EPO-D on PDGF-BB-induced VSMC proliferation.**

|  |  | **Treatment time** | | |
| --- | --- | --- | --- | --- |
| **Compound** | **Concentration (nM)** | **24h** | **48h** | **72h** |
| **EPO-B** | 0.1 | 3.2 | 37.2 | 71.3 |
|  | 1 | 29.9 | 50.1 | 78.0 |
|  | 10 | 54.4 | 61.6 | 79.7 |
|  | 25 | 54.3 | 63.7 | 81.1 |
|  | 50 | 55.1 | 67.6 | 81.4 |
|  | 100 | 49.1 | 62.3 | 81.4 |
| **EPO-D** | 0.1 | 36.3 | 59.0 | 78.5 |
|  | 1 | 28.5 | 56.9 | 78.4 |
|  | 10 | 22.6 | 60.8 | 77.9 |
|  | 25 | 30.5 | 59.8 | 75.7 |
|  | 50 | 37.2 | 57.9 | 74.5 |
|  | 100 | 26.5 | 47.1 | 69.7 |
